# Ordinary care in extraordinary times

**DOI:** 10.1017/ipm.2020.103

**Published:** 2020-09-11

**Authors:** S. Patel, A. Gannon, M. Cryan, N. McDonnell, S. Rafiq, D. Adamis, C. Dolan, G. McCarthy

**Affiliations:** 1 Sligo/Leitrim Mental Health Services for Older Persons, Sligo, Republic of Ireland; 2 School of Medicine, NUIG, Sligo, Republic of Ireland

**Keywords:** COVID-19, coronavirus, mobile tablets, nursing homes, older persons, technology

## Abstract

The Coronavirus Disease 2019 (COVID-19) has accounted for more than 25 000 cases in Ireland with approximately 28% of the clusters in nursing homes as of June 2020. The older population is the most vulnerable to serious complications from this illness and over 90% of deaths due to COVID-19 to date have been in patients over the age of 65. Continuing to provide routine care within nursing homes in these challenging times is an essential part of ensuring that presentations to hospitals for non-essential reasons are minimized. In this article, we describe a project being undertaken by a rural Psychiatry of Old Age Service in the northwest of Ireland. We aim to provide ordinary care in extraordinary times by using mobile tablets within the nursing homes and long-stay facilities in our region for remote video consultations during the COVID-19 crisis.

## Introduction

There have been more than 25,000 confirmed cases of Coronavirus Disease 2019 (COVID-19) in Ireland as of June 2020. Of these, those aged 65 and over account for approximately 25% of the cases (HPSC, 2020). Much of the focus of preparations for COVID-19 had been on increasing the resources and supports to hospitals and ICUs to reduce the projected burden on hospitals. However, as the crisis has evolved, it has become clearer that nursing homes and long-stay facilities with their older residents are also at the epicenter of this pandemic. Indeed, of the 892 clusters reported in Ireland to date, 257 (28.8%) were in nursing homes and an additional 29 (3.3%) were in community hospitals or long-stay units (HPSC, 2020). The increased risk of COVID-19 infection transmission in these settings may be attributed to an increased requirement for support with personal care, difficulties in social isolation, and increased comorbidities including cognitive impairment which could limit insight (McMichael *et al.* 2020).

The older population is the most severely affected by the COVID-19 virus with increased complications and risk of death (Wang *et al*. 2020). More than 90% of the deaths due to COVID-19 in Ireland to date have been in patients aged 65 years or older (HPSC, 2020). Long-term care residents have accounted for greater than 60% of total deaths related to COVID-19 in some European countries (ECDC, 2020). In addition to the development of life-threatening respiratory symptoms, the older patient is also at increased risk of developing non-cognitive symptoms in the context of stress, dementia, or delirium on a background of COVID-19 infection (Armitage et al. 2020; Wang *et al*. 2020). Even in the absence of a potential COVID-19 infection, they may have multiple comorbidities including cognitive impairment and associated non-cognitive symptoms which require increased psychiatric and medical support (Moore *et al*. 2014; Jørgensen *et al*. 2018). Prompt address is required to support patients, help reduce the burden of care on staff, and help reduce hospitalizations.

The nursing home COVID-19 clusters have highlighted the need to reduce footfall in these facilities in order to limit exposure in this vulnerable population and restrictions are now in place. However, life goes on and routine psychiatric and medical care is still required. Indeed, the rapidly evolving COVID-19 pandemic has also highlighted the need to develop alternative means of assessing and providing routine care remotely in this vulnerable population.

In this article, we outline a project that we are undertaking within the northwest of Ireland to facilitate routine psychiatric and medical care in the local nursing homes and long-stay facilities. Our aim is to use mobile tablet technology to support service provision to our most vulnerable population during the period of this crisis, to assess the acceptability, feasibility, and impact of this technology, and to continue to provide ordinary care in extraordinary times.

## Method

This project is being undertaken through the Psychiatry of Old Age Service covering the Sligo/Leitrim region in the northwest of Ireland. We have recruited 16 nursing homes and other long-stay facilities within the Sligo/Leitrim region with a total bed population of *n* = 657. Ethical approval for this project has been obtained through the Sligo University Hospital Research and Education Foundation Ethics Committee.

We are loaning a mobile tablet and Wi-Fi hub to each of these facilities if they are not already available within the facility, for the duration of the COVID-19 crisis. The mobile tablets and hubs were sourced through donations and through a local seed fund grant for a second associated project that is currently on hold due to the COVID-19 restrictions. Each of the mobile tablets have been equipped with Skype video communication software to facilitate remote video consultations (Skype, 2020). This is a Health Service Executive (HSE)-approved communication medium (HSE, 2018). This equipment will be on loan for the duration of the COVID-19 crisis and can be used by all medical professionals who are involved in the care of the residents of these facilities.

### Pathway of care

All GPs overseeing the primary care of the residents of these facilities are being notified and invited to utilize the equipment in their own routine care. We have also met with the Palliative Care and Geriatrics services to discuss these measures and provide a collaborative secondary care approach. A list of the Skype contact information for these facilities has been compiled and disseminated to the appropriate services.

The Palliative Care and Psychiatry of Old Age services have provided a pathway of care and an additional point of contact for referrals from these facilities for the use of the mobile tablet for their assessments. Any patient identified by the staff of the long-stay facility as requiring further psychiatric or medical input will initially be referred to the GP who could then assess the patient and refer the patient appropriately for psychiatric or palliative care input if indicated. Normal care pathways will be followed. Tablets will also be available at the hub of these secondary services that are providing specialist care. Remote video consultations using the tablets will then be arranged with the facility by these services if indicated.

### Consent and exclusion criteria

Although all efforts to obtain informed consent from the patients will be made for these video consultations, conditions such as dementia and delirium may limit the capacity to provide informed consent. Some patients who lack capacity may need to be assessed (in their best interests) to provide urgent medical or psychiatric care.

For inclusion in the research study, informed consent will be sought from all patient and staff participants of the remote video consultations. All staff who refuse to participate in the study will be excluded.

Although many patients may lack the capacity to consent to the study, the rationale for the study will be explained to all patients. We will exclude any patients who have the capacity and refuse to participate in this study. We will also exclude patients without capacity whose next of kin or representative refuses participation on their behalf or are not available to consent. This is in accordance with the local ethics committee and previous research on patients who lack capacity (Adamis *et al*. 2010; Sweet *et al*. 2014).

### Evaluation

This project will be evaluated to assess the impact and feasibility of using mobile tablets in long-stay facilities during the COVID-19 crisis. All patients residing at 1 of the 16 long-stay facilities and undergoing a Psychiatry of Old Age assessment using the mobile tablets will be recruited for this study. Additionally, all staff and key workers involved in these assessments will be recruited.

A record of each consultation will be kept as per HSE guidelines in the patient’s medical chart. Data on each psychiatry consultation will also be included in the Psychiatry of Old Age patient database for service provision, continuity of care, and metrics.

We will collect information on patient age + gender, diagnosis, reason for assessment, and outcome including medication advice. We will also gather data on the rates of referrals, hospitalizations, overall antipsychotic, and benzodiazepine use in this population during the study period, and compare with previous data from our service. Data on COVID-19 infections will also be collected to assess the impact of its presence on the perceptions of the use of remote consultations.

A brief patient/keyworker and assessing staff satisfaction survey will be offered at the end of each consultation to assess the impact of the video consultations and evaluation of the service. We were unable to find a standardized instrument for this survey but developed one for our service evaluation needs after reviewing previous similar qualitative studies.

The assessing staff survey will evaluate the following (rated on a 5-point Likert scale): (a) quality of transmission, (b) effectiveness in diagnosing patient, (c) effectiveness in developing a management plan, and (d) overall satisfaction.

The keyworker/patient survey will evaluate the following (Y/N) questions: (a) the clinician was able to understand me clearly, (b) I was able to adequately communicate with the clinician, (c) I found this mode of communication useful, and (d) I was satisfied with this consultation.

Open-ended verbal feedback regarding this method of remote assessment will also be obtained from all participants at the end of each video consultation and documented on the survey instrument.

Additionally, further qualitative feedback will be collected from all GPs, staff, and patients at the end of the study period. This will be obtained using focus groups via technology and one-on-one interview with open-ended questions to elicit the impact of the use of this technology, its comparison to face-to-face assessments, any challenges identified, and the feasibility of this form of patient engagement in long-stay facilities.

### Analysis

Qualitative data will be assessed using thematic analysis at the end of the study period. All quantitative analyses will be completed using statistical software SPSS Version 26.

## Discussion

The COVID-19 pandemic has led to a fundamental change in how we provide health care in Ireland and the world. Much focus has been on increasing supports to hospitals to reduce the burden on services. However, it is also imperative that we continue to provide routine care within these challenging circumstances to vulnerable populations in the community. The continuation of routine care during the current restrictions is particularly relevant for long-stay facility residents who have a high rate of comorbidities and require input from several medical services, which support the older persons (Moore *et al*. 2014; Jørgensen *et al*. 2018). The care needs of these residents are also likely to increase in the context of behavioural, psychological, and medical complications of COVID-19 (Armitage and Nellums et al. 2020; Wang *et al*. 2020). Recommendations on dementia care, particularly in care settings, include the provision of mental health care using a collaborative approach with dementia experts as leads (AGS, 2020; O’Neill, 2020; Wang *et al*. 2020).

Ireland and many other countries have been slow to incorporate telemedicine into routine care (Kruse et al. 2018). However, in just a few weeks, remote telephone clinics are now commonplace and virtual clinics allowing video communication are in the process of being rolled out. Indeed, the use of telemedicine has been shown to be helpful during times of crises such as outbreaks (Ohannessian, 2014). Videoconferencing has also been previously been shown to be feasible in managing the medical and psychiatric care needs of older adults in care homes (Newbould *et al*. 2017). With the current restrictions on travel and entry into long-stay facilities, this is a particularly relevant means of providing care. However, the majority of the long-stay public facilities only have access to desktop computers. The lack of mobile equipment could limit virtual assessments of long-stay facility residents with limited mobility or cognitive impairment. This is particularly relevant when the imperative is to continue to maintain social distancing and limit exposure to COVID-19. Mobile tablets that can be brought to the patient and cleaned after each use can provide a solution to these challenges and allow for assessments of these patients while limiting their exposure.

Social distancing measures have also led to increased social isolation for both the residents and the staff working in these facilities (HPSC, 2020; O’Neill, 2020). Regular social activities that facilitate distraction but increase congregation in small spaces have also ceased (HPSC, 2020). Wang *et al*. (2020) noted increased signs of anxiety and burnout in staff after a month of lockdown due to the fear of infection and worries about the residents’ condition in China. It is imperative we support the staff in order to prevent a similar situation. Installing mobile tablets in these facilities would not only allow for medical care and provide support to staff in managing challenging patients rapidly but also allow for more social communication with family members for patients who are struggling or rapidly deteriorating. They can play a vital role in the end-of-life care and links with family.

In setting up this remote service, we have come across a number of challenges that we wish to highlight for those who may consider setting up similar projects in their own service. In order to develop a collaborative approach to care in this patient population, we engaged with GPs, Palliative Care, and Geriatric Medicine. We arranged a meeting between our service, Palliative Care, and the Geriatric Medicine service leads to discuss the project and invite them to engage. There were some concerns raised initially that the project could increase direct referrals from nursing homes to secondary care services, rather than ensuring that GPs continue to provide primary care as is the case currently. Additionally, there was some concern that this would place an additional burden on the Geriatric Medicine services who are based in the acute hospital but who were anticipating a high stress on inpatient services due to COVID-19 admissions. In order to mitigate this, we highlighted that this project was to allow us to facilitate routine care. We developed a care pathway to be distributed to the nursing homes involving Psychiatry of Old Age and Palliative Care that highlighted that a GP referral would be required. Geriatric Medicine were happy to support these two services internally with any issues that may arise and continue to provide routine care in their own remote clinics along with managing hospital presentations. We also engaged all GPs involved with these facilities by inviting them to use the tablets for their own assessments and highlighting the referral pathway with a single point of contact for the Psychiatry of Old Age and Palliative Care services.

The second challenge was through the information technology (IT) services, which needed to ensure patient confidentiality and data protection issues. This delayed the mobilization of the tablets slightly but obtaining ethical approval was essential in allowing this project to go ahead from the IT perspective. There was also a large disparity between the private and public care facilities in terms of resources. Most private nursing homes we approached were already set up with mobile tablets and Wi-Fi and were willing to use this equipment for remote consultations. However, we needed to provide all public facilities on our list with the correct equipment. Regarding funding, we were fortunate to have tablets available from a second project which had received funding from a local seed grant and was on hold due to the current restrictions. However, we were also able to obtain more tablets through donations from a large corporation and a local hardware store. We also sought donations for the Wi-Fi hubs and they have been provided by a major network for 6 months. HSE phones were not compatible to linking for Internet with the tablets so the Wi-Fi hubs were invaluable in setting up the facilities with the tablets.

The COVID-19 restrictions have highlighted to us the need to improve funding for technology-based measures within the health service in Ireland. As the HSE develops its mobile technology resources, other platforms can be used such as Attend Anywhere or BlueEye Direct Video Service (Attend Anywhere, 2020; HSE Digital Transformation, 2020). Attend Anywhere has been used in Victoria, Australia for the past 18 years and more recently in the National Health Service (NHS) in the United Kingdom (UK) (TEC 2020; Attend Anywhere 2020). Other applications such as Zoom have increased in popularity but there have been security concerns regarding Zoom (Oliver, 2020; Zoom, 2020). Microsoft Teams has been widely used in the education sector and our Palliative Care colleagues as well as Mental Health Commission have been using WhatsApp video call which has end-to-end encryption (Giordano *et al*. 2017; Martin and Tapp, 2019; Microsoft, 2020; WhatsApp, 2020).

This project has a number of limitations. A high proportion of residents in long-stay facilities have cognitive impairment which may limit their ability to participate in video consultations. Older people may be less familiar with the use of new technologies. However, even a visual examination from a distance of a person who is in distress or has visible medical difficulties would allow for the prompt management of their issues. Other potential limitations include the familiarity of various staff with using the equipment although support will be provided and the strength of the Wi-Fi signal in remote settings which may limit the assessments. Kruse et al. (2018) in their systematic review identified a range of barriers to telehealth very relevant to our study including poor broadband, age-related barriers, and resistance to change.

However, there is a significant need to establish a means of providing medical and psychiatric to support the vulnerable older population in the long-stay facilities. This project will allow us to assess the feasibility of using mobile tablets in providing this support and this could potentially be incorporated as part of routine care in the future as a preventative measure for future crises.

## Conclusion

Nursing homes and other long-stay facilities with their vulnerable older residents pose a significant challenge in providing routine care with the current necessary restrictions in place due to COVID-19. However, it is imperative that such care continues and the installation of mobile technology within these facilities provides a solution to these challenges. Further evaluation will allow us to develop new ways of remote working that can improve and facilitate care in a routine way going forward. This pandemic has forced us to address some of the barriers in a practical and meaningful way allowing care to continue where it is needed most.
